# Impact of subtype C-specific amino acid variants on HIV-1 Tat-TAR interaction: insights from molecular modelling and dynamics

**DOI:** 10.1186/s12985-024-02419-6

**Published:** 2024-06-25

**Authors:** Piwai T. Gotora, Keaghan Brown, Darius R. Martin, Rencia van der Sluis, Ruben Cloete, Monray E. Williams

**Affiliations:** 1 Human Metabolomics, North-West University, Potchefstroom, South Africa; 2 South African Medical Research Council Bioinformatics Unit, South African National Bioinformatics Institute, University of the Western Cape, Bellville, South Africa; 3 Department of Science and Innovation/Mintek Nanotechnology Innovation Centre, Biolabels Node, University of the Western Cape, Bellville, South Africa

**Keywords:** HIV-associated neurocognitive disorders, Tat polymorphisms, Molecular modelling, Molecular docking, Molecular dynamic simulation, Subtype B, Subtype C

## Abstract

**Background:**

HIV-1 produces Tat, a crucial protein for transcription, viral replication, and CNS neurotoxicity. Tat interacts with TAR, enhancing HIV reverse transcription. Subtype C Tat variants (C31S, R57S, Q63E) are associated with reduced transactivation and neurovirulence compared to subtype B. However, their precise impact on Tat-TAR binding is unclear. This study investigates how these substitutions affect Tat-TAR interaction.

**Methods:**

We utilized molecular modelling techniques, including MODELLER, to produce precise three-dimensional structures of HIV-1 Tat protein variants. We utilized Tat subtype B as the reference or wild type, and generated Tat variants to mirror those amino acid variants found in Tat subtype C. Subtype C-specific amino acid substitutions were selected based on their role in the neuropathogenesis of HIV-1. Subsequently, we conducted molecular docking of each Tat protein variant to TAR using HDOCK, followed by molecular dynamic simulations.

**Results:**

Molecular docking results indicated that Tat subtype B (TatWt) showed the highest affinity for the TAR element (-262.07), followed by TatC31S (-261.61), TatQ63E (-256.43), TatC31S/R57S/Q63E (-238.92), and TatR57S (-222.24). However, binding free energy analysis showed higher affinities for single variants TatQ63E (-349.2 ± 10.4 kcal/mol) and TatR57S (-290.0 ± 9.6 kcal/mol) compared to TatWt (-247.9 ± 27.7 kcal/mol), while TatC31S and TatC31S/R57SQ/63E showed lower values. Interactions over the protein trajectory were also higher for TatQ63E and TatR57S compared to TatWt, TatC31S, and TatC31S/R57SQ/63E, suggesting that modifying amino acids within the Arginine/Glutamine-rich region notably affects TAR interaction. Single amino acid mutations TatR57S and TatQ63E had a significant impact, while TatC31S had minimal effect. Introducing single amino acid variants from TatWt to a more representative Tat subtype C (TatC31S/R57SQ/63E) resulted in lower predicted binding affinity, consistent with previous findings.

**Conclusions:**

These identified amino acid positions likely contribute significantly to Tat-TAR interaction and the differential pathogenesis and neuropathogenesis observed between subtype B and subtype C. Additional experimental investigations should prioritize exploring the influence of these amino acid signatures on TAR binding to gain a comprehensive understanding of their impact on viral transactivation, potentially identifying them as therapeutic targets.

**Supplementary Information:**

The online version contains supplementary material available at 10.1186/s12985-024-02419-6.

## Introduction

HIV-1 is classified into types 1 and 2, with HIV-1 having evolved from non-human primate immunodeficiency viruses of Central African chimpanzees [1] and HIV-2 from West African sooty mangabeys [2]. As of 2021, an estimated 38.4 million cases of HIV were recorded worldwide, with 650 000 deaths and 1.5 million new infections [3]. HIV is mostly prevalent in countries including South Africa, Portugal, Brazil, Mexico, Peru, Spain, Germany and the United States [4]. Sub-Saharan Africa is home to only 12% of the global population, yet accounts for 71% of the global burden of HIV infection [5]. HIV-1 is grouped into four groups: M, N, O and P. The group M is distributed worldwide, and it accounts for almost 99% of the global HIV-infections [6, 7].

The Group M viruses are further subdivided into nine subtypes A-D, F-H, J and K [8]. These subtypes, also known as clades, are linked geographically or epidemiologically [9]. Two subtypes are of particular interest, namely HIV-1 subtype B (HIV-1B) and subtype C (HIV-1C) as these subtypes are responsible for the highest prevalence of HIV-1 infection and HIV-associated neurocognitive disorders (HAND). Statistically, HIV-1C represents about 15 million of the world’s HIV infected population, while the second most prevalent HIV-1B accounts for over 3 million infected individuals [10]. HIV-1C is the most prevalent HIV strain and is the predominant subtype in India and Southern Africa [10]. HIV-1B is prevalent in almost all parts of Europe and the Americas, while a diverse variety of subtypes are found in West and Central Africa [11].

One way to observe diverse clinical outcomes among HIV-1 subtypes is through variations in individual proteins [12] in particular, the HIV-1 viral Transactivator or transcription (Tat) protein. Genetic variation of HIV-1 Tat exon 1 and full-length Tat differs according to subtypes [13]. The rate of nucleotide substitution for Tat in HIV-1 subtypes B and C was 1 to 1.7 x 10^3^ substitutions per site per year [14]. A mutation rate in the range of 4.1 ± 1.7 x 10^3^ per base per cell is regarded as extremely high for any biological entity [15]. This mutation rate mentioned by Cuevas et al refers to the HIV viral DNA mutation rate in general resulting from the host cystidine deaminases which induce mutations in the viral DNA as a defence mechanism. However, Tat variants may differ in their capacity to activate viral transcription once it becomes engaged in transactivation of the long terminal repeat (LTR) [16].

Tat is one of the first proteins produced during viral replication and plays an important role by transactivation of the promoter [17]. HIV-1 Tat triggers efficient RNA chain elongation by binding to the transactivation response (TAR) element forming an initial portion of the HIV-1 transcript. The interaction of Tat with the TAR element is critical for enhancing the processivity of RNA polymerase II elongation complexes that initiate at the HIV-1 LTR transcriptional promoter [18] and vital for virus replication [19]. The Tat protein is subdivided into discrete segments (N-terminal, cysteine-rich, core, basic, glutamine-rich and C-terminal domains) of which the basic domain (47–59) is essential for binding to TAR [20, 21]. It has been argued that Tat subtype C has a higher ordered structure and is less flexible compared to Tat subtype B [22], thus making the Tat-TAR complex of Tat subtype C much more stable than the complex with Tat subtype B, and increasing NF-kB activation, promoting higher transactivation by Tat subtype C [23]. Further, a study has also demonstrated the inverse with a greater transactivation capacity for Tat subtype B [22]. Specific Tat amino acid substitutions between HIV subtypes have more specifically shown to influence Tat-TAR binding. In a recent review done by our group, we have highlighted that (1) both N-terminal and C-terminal amino acids outside the basic domain (47–59) may be important in increasing Tat-TAR binding affinity, and (2) substitution of the amino acids Lysine and Arginine (47–59) resulted in a reduction in binding affinity to TAR observed in Tat subtype B [20]. This indicates the relevance of the specific amnio acids in the biological activity of Tat.

In addition to Tat being a multi-functional regulatory protein involved in transcriptional enhancement by binding TAR, Tat also plays a crucial role in causing neurotoxicity and dysfunction in the central nervous system (CNS) [24]. The HIV tat protein, together with glycoprotein (gp) 120 decrease glial and synaptic glutamate uptake, stimulating the release of glutamate from nerve ending, phosphorylating glutamate receptors, thus potentiating the toxicity of neurotransmitters [24]. Furthermore, subtype variation has also been shown to influence Tat’s neurotoxicity. *In vitro* studies have found that Tat subtype B is more potent in neurotoxicity than Tat subtype C when inducing neuronal death [25]. The observed variations are associated with specific Tat amino acid substitutions [26]. In subtype C, polymorphisms within the TAR binding domain including serine substitutions at residue 31 and residue 57 and glutamate substitution at residue 63, can influence neurotoxicity and neurocognitive outcome in PLWH [26]. The lesser neurotoxicity of Tat subtype C compared to Tat subtype B might be attributed to the mutation found at cysteine 31 which is vital in mediating persistent excitation of N-methyl-D-aspartate receptor (NMDAR) [27]. The R57 signature in Tat subtype B is crucial for transactivation and the level of neuroinflammation by Tat subtype B was significantly reduced by the R57S substitution which is present in Tat subtype C [28]. Furthermore, a Q63E mutation present in Tat subtype C was shown to contribute to higher transcriptional activation in human CD4 T cells [29].

In a recent scoping review conducted by our group, we examined all studies investigating Tat-TAR interaction using various molecular techniques [20], including but not limited to surface plasmon resonance [30], electromobility shift assay [31], gel electrophoresis and circular dichroism [32]. However, to date, only n = 23 studies have evaluated the influence of amino acid substitutions on Tat-TAR interaction, despite this being a fundamentally important aspect potentially contributing to the observed differential pathogenesis between subtypes. Furthermore, a significant majority of these studies were conducted before the year 2000 (53%) [20]. To the best of our knowledge, only two computational studies have been conducted on this topic in 2017 and 2022, respectively [33, 34]. Given the advancements in research techniques, particularly in viral genome sequencing of HIV-1 and molecular docking simulations, there is a pressing need for more recent investigations. Importantly, to date, no study, at either the *in silico* or molecular levels, has conducted a comparison of subtype-specific Tat variations and their impact on Tat-TAR binding.

In a previous study by our group, we compared Tat subtype B and subtype C binding to TAR. Our findings indicated that Tat subtype B exhibited a higher affinity for the TAR RNA element compared to Tat subtype C. This conclusion was based on several factors, including a higher docking score of -187.37, a higher binding free energy value of -9834.63 ± 216.17 kJ/mol, and a greater number of protein-nucleotide interactions (26 interactions). Additionally, it was observed that Tat subtype B displayed more flexible regions when bound to the TAR element, which could potentially account for its stronger affinity to TAR [34]. However, it is important to note that the previous study only compared variations between Tat subtypes, without specifically examining the potential impact of individual amino acid substitutions. Furthermore, both our previous investigation [34] and another *in silico* study [33] utilized truncated versions of the Tat protein. However, it has been established that the full-length Tat protein holds greater biological relevance for understanding Tat function [35, 36].

Therefore, to further explore the initial findings, our objective was to investigate the impact of specific amino acid substitutions in the full length Tat (known to be involved in neuropathogenesis) on TAR binding. These substitutions include C31S, R57S, Q63E, and a combination of these three (C31S/R57S/Q63E). The main aim was to determine which of these substitutions would exert the most significant effect on TAR binding by using an *in silico* approach including molecular modelling, docking and simulation studies. We hypothesized that all investigated subtype C-specific amino acid substitutions may reduce the predicted binding affinity to TAR compared to the reference Tat subtype B-specific amino acids. Furthermore, we anticipated that the most significant effects will be noted for amino acid substitutions within the Tat basic domain. By examining the influence of these specific amino acid substitutions on Tat-TAR binding, we hope to shed light on the potential impact of subtype-specific amino acid variations on the dynamics of Tat-TAR binding and contribute to a better understanding of HIV infection in diverse populations.

## Materials and methods

### Retrieval of Tat subtype B sequence and TAR RNA structure

The HIV-1 subtype B Tat wildtype (TatWt) protein sequence was obtained from the Universal Protein Databases (Taxon ID: 11696) (https://www.uniprot.org/) (Version release 2023_04). The Tat subtype B (Isolate MN, P05905) was used as it contained the sequence variation which is related to the differential HIV-1 neuropathogenesis [26]. Tat interacts with various host factors that contribute to its binding affinity with TAR. In our study, we specifically focused on TAR as the major interacting partner to investigate how Tat variants lead to diverse levels of binding. The experimentally determined 3D structure of the TAR RNA was obtained by downloading the corresponding file from the Protein Data Bank (PDB ID: 1ANR). In this study, a specific region of TAR RNA spanning from nucleotides 17 to 45 was chosen for subsequent docking studies. This region was deemed sufficient to assess Tat-TAR binding, consistent with methodologies employed in prior computational studies [33, 34]. This region includes the bulge region (+23 to +25) known to contain nucleotides that interact with the HIV-1 Tat protein [37].

### Three-dimensional protein structure prediction using MODELLER v10.4

**Fig. 1:**
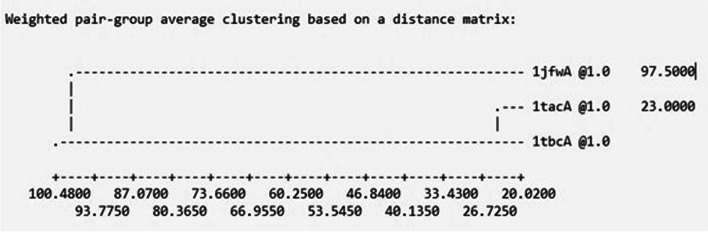
Clustering tree representing template structural similarity

MODELLER (v 10.4) is a free for academic use, standalone software tool widely used for the homology or comparative modelling of the three-dimensional structures of protein molecules that allows for user input and modification [38]. This tool utilizes a set of standard Python libraries to process a user-provided protein sequence in the PIR format. The initial template search is performed by the ‘Proile.build()’ command which initializes a Python ‘environment’ for the particular modelling build. This creates a MODELLER-built sequence database, and filters sequences less than 30 and greater than 4000 amino acids in length. The script then reads both the generated binary database and target sequence to perform an alignment for template identification with standard BLOSUM62 similarity matrix parameters. The target TatWt sequence was used to search the protein data bank profile database and only four templates (1JFW, 1K5K, 1TAC, 1TBC) were identified by executing the build_profile.py routine in MODELLER. All four templates were downloaded and selected for creating a multiple sequence alignment due to their high sequence identities: 1JFW at 91%, 1K5K at 72%, 1TAC at 65%, and 1TBC at 75%, with all E-values equal to 0, indicating suitability for modelling. Subsequently, the 'Alignment.compare_structure()' routine, which implements "malign3d" and is part of the compare.py script, was executed. This routine performs an iterative least-squares superposition of the four 3D structures, generating an alignment and dendrogram based on both sequence and structural similarities, as depicted in Fig. 1.

The TatWt protein sequence was aligned with the template sequence 1JFW, because it showed the highest sequence similarity and lowest crystallographic resolution (1Å). This specific template encompasses 11 different conformations, all the different conformations were considered in the model generation process as it generates an averaged conformation accounting for muti-conformational states of the structure. An alignment was created between the target sequence and the template sequence, and the resulting output file was passed to the MODELLER 'AutoModel' class. This class was utilized to generate a set of five potential 3D models, each with coordinates in the PDB format. These models were based on the alignment file and the 3D structures of the templates. Additionally, MODELLER calculates Discrete Optimized Protein Energy (DOPE) assessment scores and GA341 scores for the predicted protein models. The DOPE score is a pairwise atomistic statistical potential score that is used to distinguish favourable protein models from poorly predicted protein models [39]. Usually, protein models with lower DOPE score values are close to their native state [39]. GA341 scores is a fold assessment score used to determine if the correct template was selected for model building and ranges between 0 (worst) to 1 (best) [40]. Following this, a DOPE assessment plot was generated to compare scores between the template and the selected model to determine any regions of high energy corresponding to unresolved loop regions. The final protein model with the lowest DOPE score and a GA341 value close to 1 was selected for further analysis.

### Energy minimization

Input files were generated with CHARMM-GUI web servers [41, 42] with the predicted TatWt protein structure (section "Three-dimensional protein structure prediction using MODELLER v10.4") undergoing energy minimization (EM), optimizing the arrangement of atoms within the three-dimensional structure to achieve the most energetically favourable state for the protein [43]. EM was performed using the CHARMM36M force field within the GROMACS package with 50,000 steps of steepest descents to eliminate any steric overlaps.

### Predicted protein structure quality assessment

After energy minimization, the TatWt protein structure was submitted to the Structure Analysis and Verification Server (SAVES v.6) web server, accessible at: https://saves.mbi.ucla.edu. This web server offers a range of tools, including ERRAT, which is utilized to identify areas where errors resulted in random atom distributions; Verify3D, employed to analyse the compatibility of the atomic model (3D) with its amino acid sequence (1D); and PROCHECK, which assesses the stereochemical quality of the protein structure(s). Each tool has specific criteria for evaluation. A score of 50 and above for ERRAT is considered a pass, while a score of 50 and below is considered a failure. For Verify3D, a score above and close to 80% is deemed a pass, whereas scores below 70% are considered a less than optimal protein structure. PROCHECK analyses include the generation of a Ramachandran plot that calculates the distribution of torsion angles (φ and ψ) of C-alpha residues in a protein structure and if more than 90% of residues satisfy the dihedral angle distribution [44].

Lastly, the TatWt minimized protein model was passed to the ProSA webserver which compared 3D models to experimentally resolved structures based on the z-scores of the X-ray or crystallographic resolution techniques [45]. The z-score indicates overall model quality and measures the deviation of the total energy of the structure with respect to an energy distribution derived from random conformations. Z-scores outside a range characteristic for native proteins indicate erroneous structures [45]. The 1JFW template was superimposed onto the predicted protein model using PyMOL (v2.5.5) align command. RMSD values lower than 1.5Å indicate a high structural similarity between a pair of structures suggesting homology [46].

### Structural quality assessment

We utilized Tat subtype B structure as the reference or wild type, and generated Tat variants to mirror those found in Tat subtype C. Therefore, once the TatWt model passed all quality checks, the variant positions were introduced into the Wt structure generating TatC31S, TatR57S, TatQ63E, and TatC31S/R57S/Q63E variant structures using the initial phase of the Automated Mutation Introduction and Analysis (AMIA) pipeline. These amino acid variants are predominantly found in subtype C Tat, with prevalences of C31S = 82%, R57S = 74%, and Q63E = 80% in Brazilian subtype C cases [47]. These subtype C-specific mutations were selected for investigation based on their role in the neuropathogenesis of HIV-1 [34]. This pipeline allows the user to automatically introduce residue substitutions at specific positions within the static protein structure and subsequently automatically analyses the trajectory statistics of the simulations of these systems, and the pipeline is accessible at: https://github.com/kbrown3687524/amia.git. Subsequently, the TatWt and Tat variant structures were used in docking studies of the TAR element to the respective structures.

### Molecular docking using HDOCK

Molecular docking is a method used to predict the binding affinity between protein-ligand, protein-DNA/RNA and protein-protein/peptide complexes [48]. The HDOCK webserver (http://hdock.phys.hust.edu.cn/) is a highly integrated suite of homology search, template-based modelling, structure prediction, macromolecular docking, biological information incorporation and job management for robust and fast protein–protein docking. It distinguishes itself from similar docking servers in its ability to support amino acid sequences as input and a hybrid docking strategy in which experimental information about the protein-protein binding site and small-angle X-ray scattering can be incorporated during the docking and post-docking processes [49]. HDOCK is also beneficial to use as it supports protein–RNA/DNA docking with an intrinsic scoring function**.** The docking scores are calculated by knowledge-based iterative scoring function ITScorePP or ITScorePR and a more negative docking score means a stronger association between the molecules. Roughly, when the confidence score is above 0.7, the two molecules would be very likely to bind; when the confidence score is between 0.5 and 0.7, the two molecules will possibly bind; when the confidence score is below 0.5, the two molecules would be unlikely to bind [49]. The 3D minimized structures of the respective Tat proteins and the TAR structure obtained from the Protein Data Bank were uploaded to the HDOCK server. The region of the Tat protein known as the basic region, comprising residues 48-58 [20, 50] and the bulge region of the TAR, spanning positions +23 to +25 [37], have been identified as the binding site for Tat-TAR interaction. These specific residues were selected as the active site residues to define the search space for the docking simulation.

### Protein- RNA interaction analysis

Furthermore, we analysed the protein-RNA interaction. We used the Protein-Ligand Interaction Profiler (PLIP), an analytical tool designed to detect and visualize relevant non-covalent protein-ligand interactions in 3D structures (https://plip-tool.biotec.tu-dresden.de/plip-web/plip/index) [51]. It functions by detecting hydrogen bonds (H-bonds), hydrophobic contacts, π-stacking, π-cation interactions, salt bridges, water bridges, metal complexes and halogen bonds between ligands and targets [52]. The cut off distance values considered in PLIP for interactions to occur between atoms were 4.1 Å for H-bonds, 4.0 Å for hydrophobic contacts, 5.5 Å for π-stacking, 6.0 Å for π-cation interactions, 5.5 Å for salt bridges, 4.1 Å for water bridges, 3.0 Å for metal complexes, and 4.0 Å for halogen bonds.

### Molecular dynamic (MD) simulation

Five systems were created, comprising the TatWt-TAR system (TatWt) and four variant systems with Tat subtype C variant residues introduced into Tat B structure at positions C31S (TatC31S), R57S (TatR57S), Q63E (TatQ63E), and a multi-variant combination system of TatC C31S, R57S, and Q63E (TatC31S/R57S/Q63E). These systems were prepared using the CHARMM-GUI webserver [42, 53]. The webserver's capabilities include generating base parameter files for various biological system types with easy modification for integration with different force fields such as CHARMM, AMBER, GROMOS, OPLS, and simulation packages like NAMD, AMBER, and GROMACS [54]. Within the CHARMM-GUI webserver interface, the Solution Builder simulator was chosen to generate the energy minimization, equilibration, and production parameter files, as there were no membrane, fibrous proteins, or lipid-like molecules present.

Each of the five systems were individually uploaded and solvated, with TIP3 water molecules in a cubic box ensuring a minimum distance of 10 Å between the protein and the edges of the box. The TIP3 water model was selected for its accurate representation of a predictive aqueous environment [55]. The solvated systems were neutralized by introducing default potassium cations (K+) and chloride anions (Cl-) at a concentration of 0.15M, utilizing the Monte Carlo ion placing method [56, 57]. This method was chosen as K+ ions are plentiful within the cell cytoplasm and do not adversely affect biomolecules [58]. The systems were neutralized with specific ion counts: 50 K^+^ and 34 Cl^-^ ions for both the TatWt and TatC31S systems, 51 K^+^ and 34 Cl^-^ ions for the TatQ63E system, 68 K^+^ and 41 Cl^-^ ions for the TatR57S system, and 53 K^+^ and 43Cl^-^ ions for the TatC31S/R57S/Q63E system. The CHARMM36M force field was utilized to generate the topology and coordinate files, chosen for its enhanced accuracy in analysing proteins, peptides, and nucleic acid molecules [41]. Each system underwent 50,000 steps of steepest descents energy minimization (EM) to eliminate any steric overlaps. Additionally, all H-bonds were constrained using the LINCS constraints algorithm [59].

After the EM, the systems underwent a two-step equilibration phase, namely, NVT (isothermal-isochoric ensemble) and NPT (isothermal-isobaric ensemble). The NVT equilibration phase ensured that the number of particles, volume, and temperature of the systems were maintained by immobilizing the solutes while allowing the solvent to move freely. The NPT equilibration phase ensured the constant maintenance of the number of particles, pressure, and temperature [60]. For the NVT equilibration, the systems were run for 100 picoseconds (ps) with a 2 femtosecond (fs) timestep, employing the V-rescale temperature-coupling method [60] with a constant coupling of 0.1 ps at 310 K to stabilize the temperature of the systems. In contrast, for the NPT equilibration, the systems were ran for 500 ps with a 2 fs timestep, utilizing the Parrinello-Rahman barostat [61] in tandem with the V-rescale thermostat under identical coupling parameters [34]. In both NVT and NPT, electrostatic forces were calculated using the Particle Mesh Ewald method [62].

The production phase for the simulations of the five systems ran under conditions of no restraints for 500 nanoseconds (ns) at an integration step of 0.002 ps [34] with the trajectory being recorded every 10ps. Trajectory analyses were performed using the AMIA Pipeline, available at: https://github.com/kbrown3687524/amia. These analyses included RMSD of the protein structures backbone atoms and TAR RNA nucleic acids, Root Mean Square Fluctuation (RMSF) of the protein residues, Radius of Gyration (Rg/Rgyr) of the protein backbone. Principal Component Analysis (PCA) of the protein backbone atoms, H-bonds as well as Ionic Interaction Analysis between the Tat and TAR element.

### Molecular mechanics poisson-boltzmann surface area (MMPBSA) calculations

The binding free energies of the TatWt and mutant systems (TatC31S, TatQ63E, TatR57S and TatC31SQ63ER57S-multi) with TAR were calculated for 1001 frames over the last 100 ns of the trajectory using the gmx_MMPBSA tool v1.4.3 [63, 64]. The MMPBSA tool is an effective strategy employed for the calculation of binding free energy of several protein-ligand complexes [65–69]. The formula for calculating binding free energy is defined as ΔG = ΔH -TΔS, with ΔG being the change in binding energy and ΔH is the change in enthalpy while T is the temperature and ΔS is the change in entropy [65–69].

## Results

### 3D Structure prediction and quality assessment

**Fig. 2:**
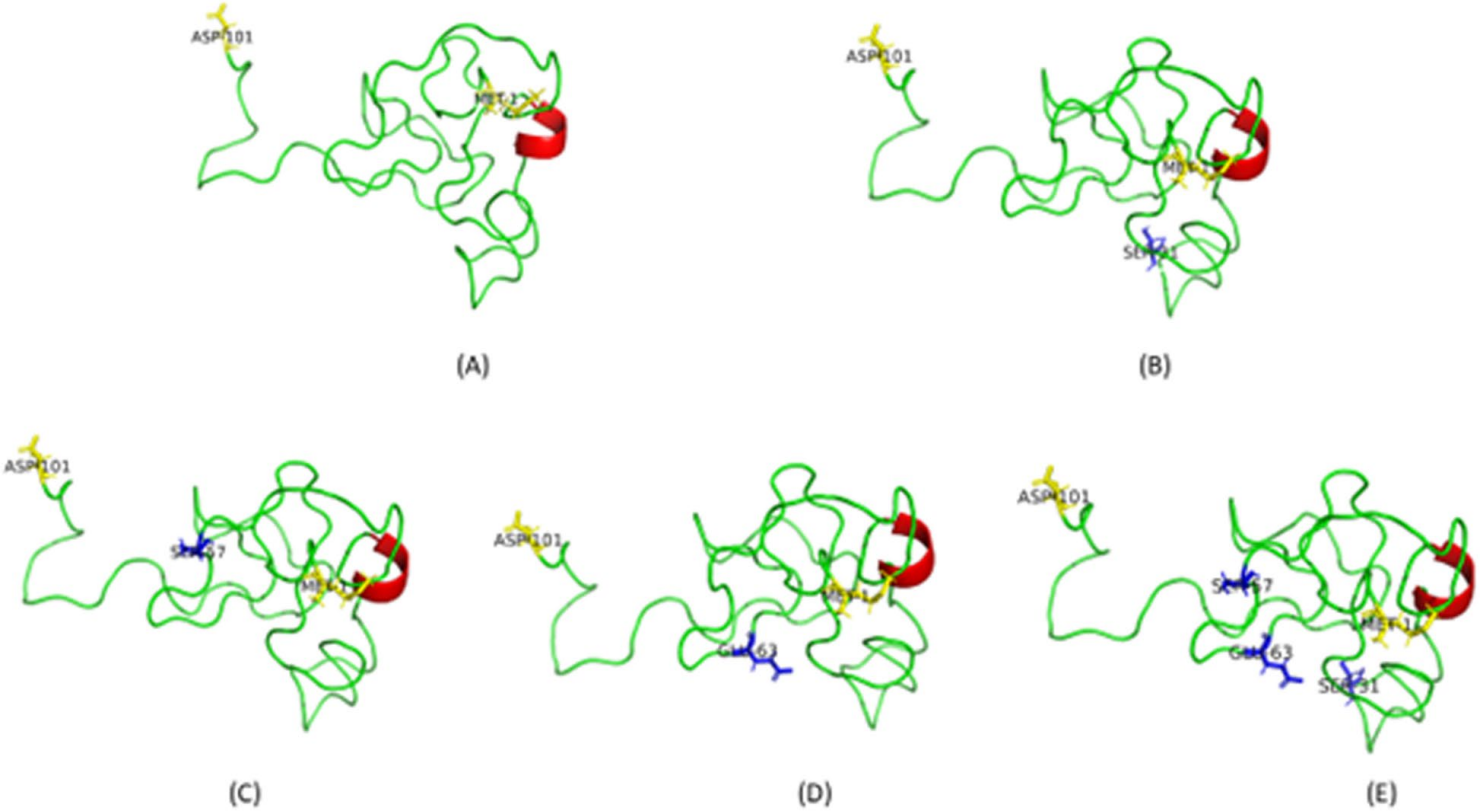
**A-E** Cartoon representation of the 3D structures for the TatWt (**A**), TatC31S (**B**), TatR57S (**C**), TatQ63E (**D**) and TatC31S/R57S.Q63E (**E**). The alpha-helical structure is indicated in red. The N-terminal Met1 and C-terminal Asp101 are shown in yellow sticks and text. The Tat amino acid variants are indicated as blue sticks for each respective model

After predicting the 3D structure using MODELLER, the software calculates its own in-built quality measures of the protein model. Based on the DOPE and GA341 scores provided by MODELLER, we selected the third model with a DOPE score of -5464.01 and a GA341 score of 1 for subsequent Quality Assessment (QA) and docking analyses. This protein model represented the TatWt structure and therefore was subjected to QA via the SAVES platform. The ERRAT score (52.2), Verify3D (76.24%), and PROCHECK summary indicated that the model met the quality criteria. ProSA-web results indicated that the model fell within the range of NMR structures with a z-score of -2.8. The RMSD was calculated by superimposing TatWt onto 1JFW, resulting in a value of 0.95 Å, indicating a high correlation between the atomic placements in the two structures. All Tat proteins were largely disordered and presented with 1 alpha helix at amino acids 38-41 (Fig. 2A, E).

### Molecular docking: HDOCK

**Fig. 3:**
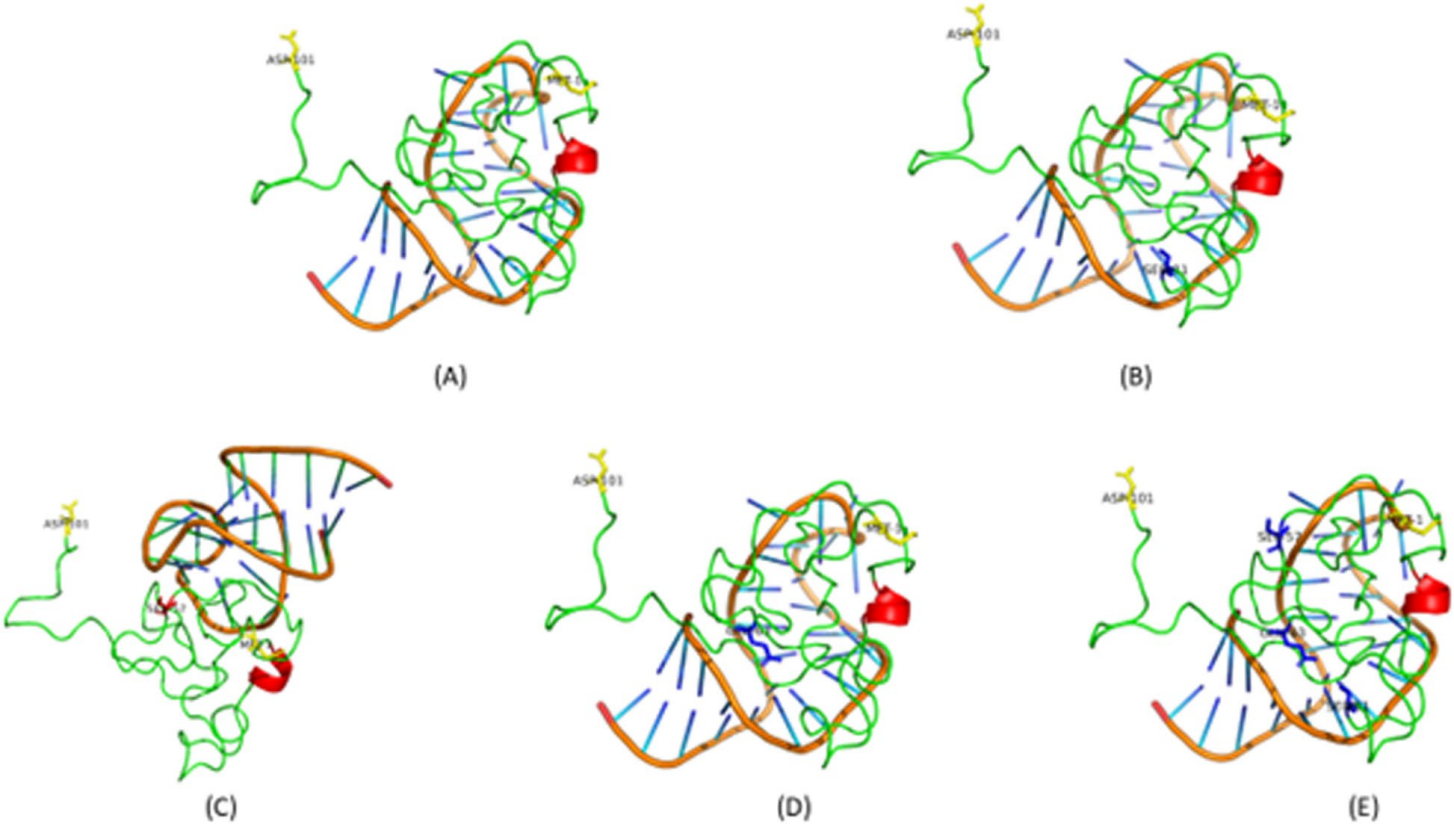
**A-E** The top predicted binding poses for TatWt and respective Tat variants against TAR. **A** TatWt, (**B**) TatC31S, (**C**) TatR57S, (**D**) TatQ63E and (**E**) TatC31S/R57S/Q63E. The alpha-helical structure is indicated in red. The N-terminal Met1 and C-terminal Asp101 are shown in yellow sticks and text. The Tat amino acid variants are indicated blue sticks and text for each respective model. R57S was the only variant amino acid interacting with TAR (Red stick, C). Molecular docking was carried out with the basic region of Tat with the bulge region of TAR

The TatWt demonstrated the highest predicted binding affinity to TAR, with a score of -262.07 (Table 1). This was followed by TatC31S (-261.61), TatQ63E (-256.43), TatC31S/R57S/Q63E (-238.92) and TatR57S (-222.24) (Table 1). In comparison to TatWt, the TatR57S amino acid variant exhibited the most substantial percentage decrease (15%) in binding affinity to TAR. This was followed by TatC31S/R57S/Q63E (8.8%), TatQ63E (2.2%), and TatC31S (0.2%). Using PLIP analysis, various types of interactions, including hydrogen bonds (H-bonds), salt bridges, and hydrophobic interactions, were identified between Tat and TAR. The number of interactions for the TatWt and Tat variants with TAR were similar, with TatC31S, TatWt and TatC31S/R57S/Q63E having 16 total interactions while TatR57S and TatQ63E having 15 and 14 interactions, respectively (Table 1). Given that H-bonds are significant contributors to intermolecular energy between molecules, it is noteworthy that TatWt, TatC31S, and TatC31S/R57S/Q63E all exhibited 13 hydrogen bond interactions. Following closely, TatQ63E demonstrated 10 hydrogen bonds, while R57S had the lowest number of hydrogen bond interactions of 8, showing strong correlation with the predicted binding affinity values (Table 1). Importantly, all binding occurred within the same binding pocket for all variant structures, besides R57S (Fig. 3C).

**Table 1:** Predicted docking score and the number and type of interactions between Tat protein and TAR RNA

**Tat variant**	**Docking Score** **(confidence score)**	**Percentage difference in predicted binding energy**	**Type of interactions**			
			**H-bonds (interacting nucleotide)**	**Salt bridges** **(Interacting nucleotide)**	**π-Cation (interacting nucleotide)**	**Hydrophobic** **(Interacting nucleotide)**
TatWt	-262.07 (0.9039)	None	GLU9 (G36), HIS33 (C24), SER68 (G36), LYS71 (G17), GLN76 (U40), GLN76 (C39), ARG78 (G26), ARG78 (G26), ARG78 (U25), ARG78 (A27), GLY79 (C24), PRO84 (A20), SER87 (C19)	LYS71 (G18)	None	VAL36 (C24), PHE38(U25)
TatC31S	-261.61 (0.9031)	0.2%	GLU9 (G36), HIS33 (C24), SER68 (G36), LYS71 (G17), GLN76 (U40), GLN76 (C39), ARG78 (G26), ARG78 (G26), ARG78 (U25), ARG78 (A27), GLY79 (C24), PRO84 (A20), SER87 (C19)	LYS71 (G18), LYS85 (A20)	None	VAL36 (C24), PHE38(U25)
TatQ63E	-256.43 (0.8937)	2.2%	HIS33 (C24), LYS71 (G17), GLN76 (U40), GLN76 (C39), ARG78 (G26), ARG78 (G26), ARG78 (U25), ARG78 (A27), GLY79 (C24), PRO84 (20A)	HIS33 (A22), LYS71 (G18)	None	VAL36 (C24), PHE38(U25)
TatC31S/R57S/Q63E	-238.92 (0.8555)	8.8%	GLU9 (G36), HIS33 (C24), LYS71 (G17), GLN76 (U40), GLN76 (C39), ARG78 (G26), ARG78 (U25), ARG78 (A27), ARG78 (U38), GLY79 (C24), PRO84 (A20), SER87 (C18), SER84 (C19)	LYS71 (U38), LYS71 (G18)	None	PHE38(U25)
TatR57S	-222.24 (0.8092)	15%	GLU9 (A27), TRP11 (G36), GLN54 (A25), ARG55 (C24), ARG56 (A27), ARG56 (C24), SER57 (A25), GLN76 (G34)	LYS12 (A35), LYS12 (G36), ARG49 (C37), LYS51 (A20), ARG53 (A20), ARG53 (G21), ARG78 (G34)	None	None

Abbreviations: *GLU* Glutamic acid, *HIS* Histidine, *LYS* Lysine, *SER* Serine *GLN* Glutamine, *ARG* Arginine, *GLY* Glycine, *PRO* Proline, *TRP* Tryptophan, *PHE* Phenylalanine, *VAL* Valine, *C* Cytosine, *G* Guanine, *U* Uracil, *A* Adenine

### MD simulations

**Fig. 4:**
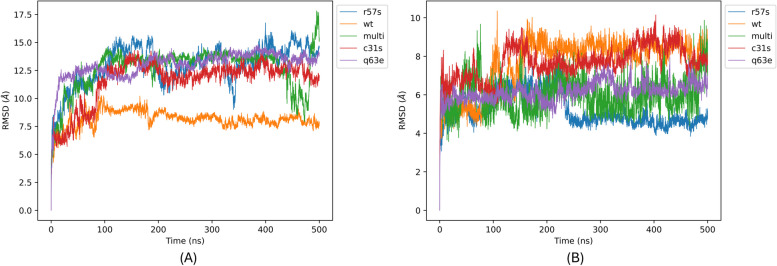
Comparison of Backbone RMSD Values for (**A**) Tat Variant and (**B**) TAR Systems. **A** depicts the RMSD plot showing the changes in Tat backbone atoms of the TatWt system (orange) and mutant systems TatC31S (red), TatR57S (blue), TatQ63E (purple), and TatC31S/Q63E/R57S (multi) (green) over the 500ns molecular dynamic simulation trajectory. All systems reported exhibited larger mean deviation values compared to the TatWt system value (orange). **B** shows the RMSD plot illustrating the changes in TAR backbone atoms of the TatWt system (orange) and mutant systems TatC31S (red), TatR57S (blue), TatQ63E (purple), and TatC31S/Q63E/R57S (multi) (green) over the 500ns molecular dynamic simulation trajectory. TatR57S (blue), TatQ63E (purple), and TatC31S/R57S/Q63E (multi) (green) exhibited lower mean deviation values compared to TatWt (orange) and TatC31S (red)

To further assess the interactions between the Tat proteins and TAR, we utilized AMIA pipeline to explore the flexibility, stability, and dynamic behaviour of the Tat-TAR interactions. The comparison of RMSD values across the trajectory offers an initial approach to investigate the structural conformations of the protein systems during the simulation. Significant differences in means and standard deviations between the wild type (Wt) and mutant variant systems provide insights into whether the substituted amino acid(s) lead to a noteworthy deviation from Wt dynamics or if the new residues cause the system to conform to Wt dynamics [70]. Analysing the backbone RMSD of the four Tat variant systems in comparison to the TatWt system revealed that TatC31S, TatR57S, TatQ63E, and TatC31S/R57S/Q63E (multi) exhibited larger mean deviation values of 1.24 ± 0.19 nm, 1.39 ± 0.18 nm, 1.37 ± 0.12 nm, and 1.34 ± 0.18 nm, respectively, compared to the TatWt system value of 0.77 ± 0.13 nm (Fig. 4A). Analysing the TAR element RMSD of each mutant system in comparison to the TatWt revealed that the TAR element for TatR57S, TatQ63E, and TatC31S/R57S/Q63E (multi) possessed lower mean deviation values of 0.52 ± 0.01 nm, 0.62 ± 0.05 nm, and 0.60 ± 0.09 nm, respectively. In contrast, system TatC31S exhibited values of 0.77 ± 0.085 nm, similar to the TatWt values of 0.77 ± 0.13 nm (Fig. 4B).

**Fig. 5:**
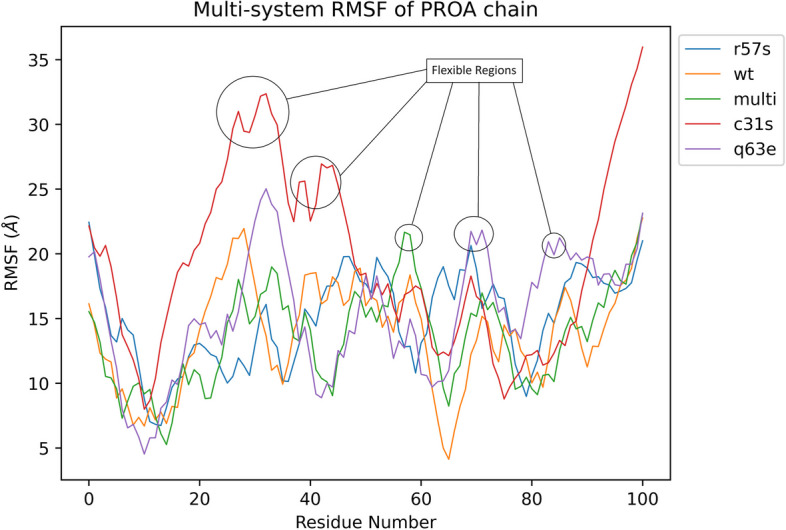
RMSF Plot of Tat Protein Residues in Variant System. This figure illustrates the Root Mean Square Fluctuation (RMSF) plot showing the changes in Tat protein residues of chain A for the TatWt system (orange) and variant systems TatC31S (red), TatQ63E (purple), TatR57S (blue), and TatC31S/Q63E/R57S (multi) (green) over the equilibrated phase (200-500ns) of the molecular dynamic simulation trajectories. The mean RMSF values for TatC31S (red), TatR57S (blue), and TatQ63E (purple) were higher than those for the TatWt system (orange), whereas the mean RMSF values for TatC31S/R57S/Q63E (multi) (green) were lower than those for the TatWt system (orange). Regions with high flexibility for the mutations of interest are circled. Notably, the C31S and Q63E systems both exhibited two regions of high flexibility that coincided with the location of the mutations

The RMSF values for each residue were calculated during the equilibrated phase of the simulation (200 – 500ns), representing the plateau region of the TatWt system [71]. The mean RMSF values for the TatC31S, TatR57S, and TatQ63E system protein residues were higher than the TatWt system each at 1.88 ± 0.67 nm, 1.51 ± 0.37 nm, 1.54 ± 0.47 nm, and 1.41 ± 0.41 nm. Conversely, the mean RMSF values for TatC31S/R57S/Q63E (1.36 ± 0.37 nm) were lower than those for the TatWt system (Fig. 5). Notably, the C31S and the Q63E systems both had 2 regions of high flexibility that coincided with the location of the mutations.

**Fig. 6:**
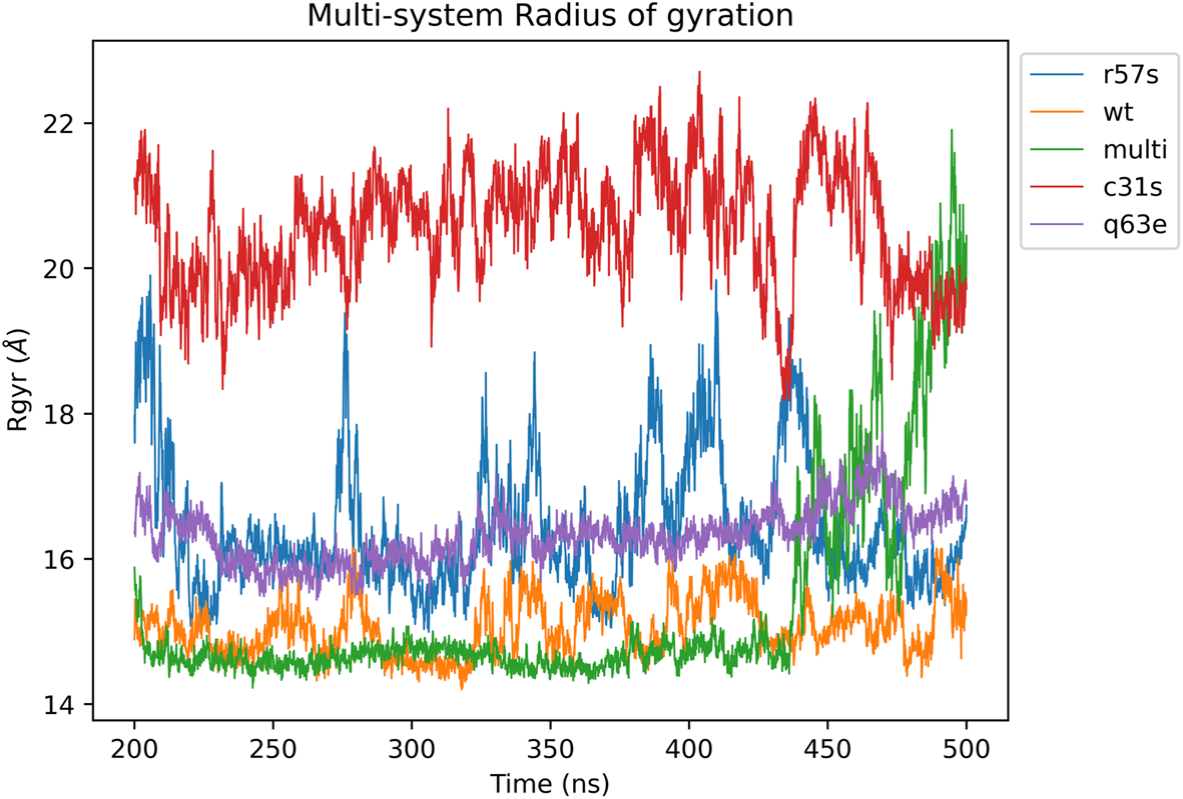
The change in Radius of gyration/Center of mass plotted for Tat backbone atoms of the TatWt system and mutant systems TatC31S, TatQ63E, TatR57S and TatC31SQ63ER57S (multi) over the equilibrated phase (200-500ns) of the molecular dynamic trajectories. Variant systems TatC31S, TatR57S, and TatQ63E showed higher Rg values compared to the TatWt system. In contrast, TatC31S/R57S/Q63E (multi) exhibited similar values

The radius of gyration (Rg/Rgyr) defines compactness of the protein structure by describing the weighted mean square distance of atoms from the centre of mass [72]. These values were calculated over the same timeframe as the RMSF analysis (200 – 500ns). Variant systems TatC31S, TatR57S, and TatQ63E showed higher Rg values at 2.07 ± 0.09 nm, 1.63 ± 0.09 nm, and 1.63 ± 0.04 nm, respectively, than the TatWt system at 1.51 ± 0.048 nm. In contrast, TatC31S/R57S/Q63E (multi) conformed to similar values at 1.52 ± 0.13 nm (Fig. 6). After 450ns, the multi variant system jumped slightly, perhaps due to increased number of mutations in the system resulting in a decrease in the compactness of the structure and possibly unfolding of the protein structure similar to the RMSD backbone values seen in last part of the simulation trajectory in Fig. 4A.

**Fig. 7:**
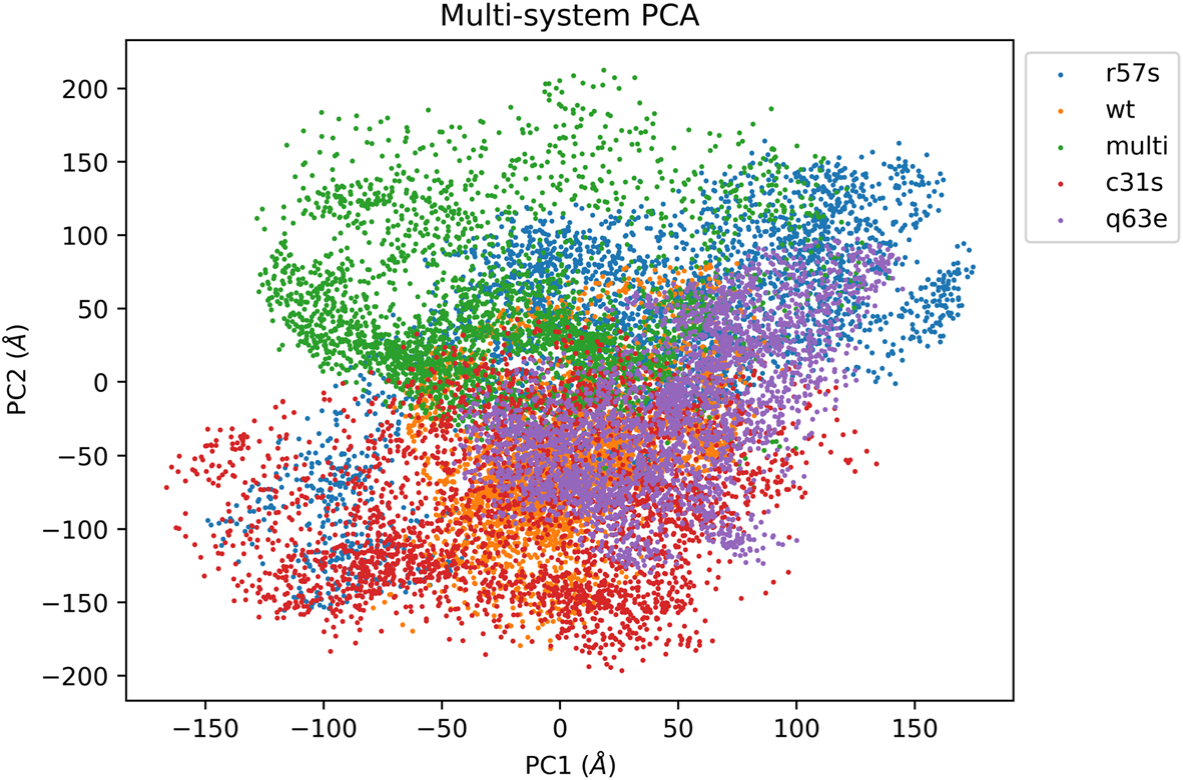
A plot of the first two Principal components (PC1 and PC2) (Å2) of the TatWt system (orange) and mutant systems TatC31S (red), TatQ63E (purple), TatR57S (blue) and TatC31SQ63ER57S (multi) (green) over the equilibrated phase (200-500ns) of the molecular dynamic simulation trajectories. The variant systems TatC31S (red) and TatR57S (blue) demonstrated significantly greater collective motion within the essential subspace, while TatQ63E (purple) exhibited a significant decrease, and the TatC31S/R57S/Q63E (multi) (green) system showed a minor decrease in collective motions, in comparison to the TatWt system (orange)

The primary motions characterizing each protein structure within their respective systems were explored employing Principal Component Analysis, a statistical method for reducing data dimensionality [73]. This method was used to calculate which of the principal components (PCs) account for the largest amount variance in the dataset. Here we considered PC1 and 2 known to account for the largest amount of variance in protein movement. The PC1 and PC2 variance were plotted in 2D space for ease of visualization. The mutant systems TatC31S and TatR57S demonstrated significantly greater collective motion within the essential subspace, while TatQ63E exhibited a significant decrease, and the TatC31S/R57S/Q63E system showed a minor decrease in collective motions, in comparison to the TatWt system. This is evident from the covariance matrix values after diagonalization, where for the TatWt system the value was 8.14 nm^2^, and for the mutant systems TatC31S, TatR57S, TatQ63E, and TatC31S/R57S/Q63E, the values were 14.75 nm^2^, 13.8 nm^2^, 4.71 nm^2^, and 9.2 nm^2^, respectively, as depicted in the PCA plot (Fig. 7).

**Fig. 8:**
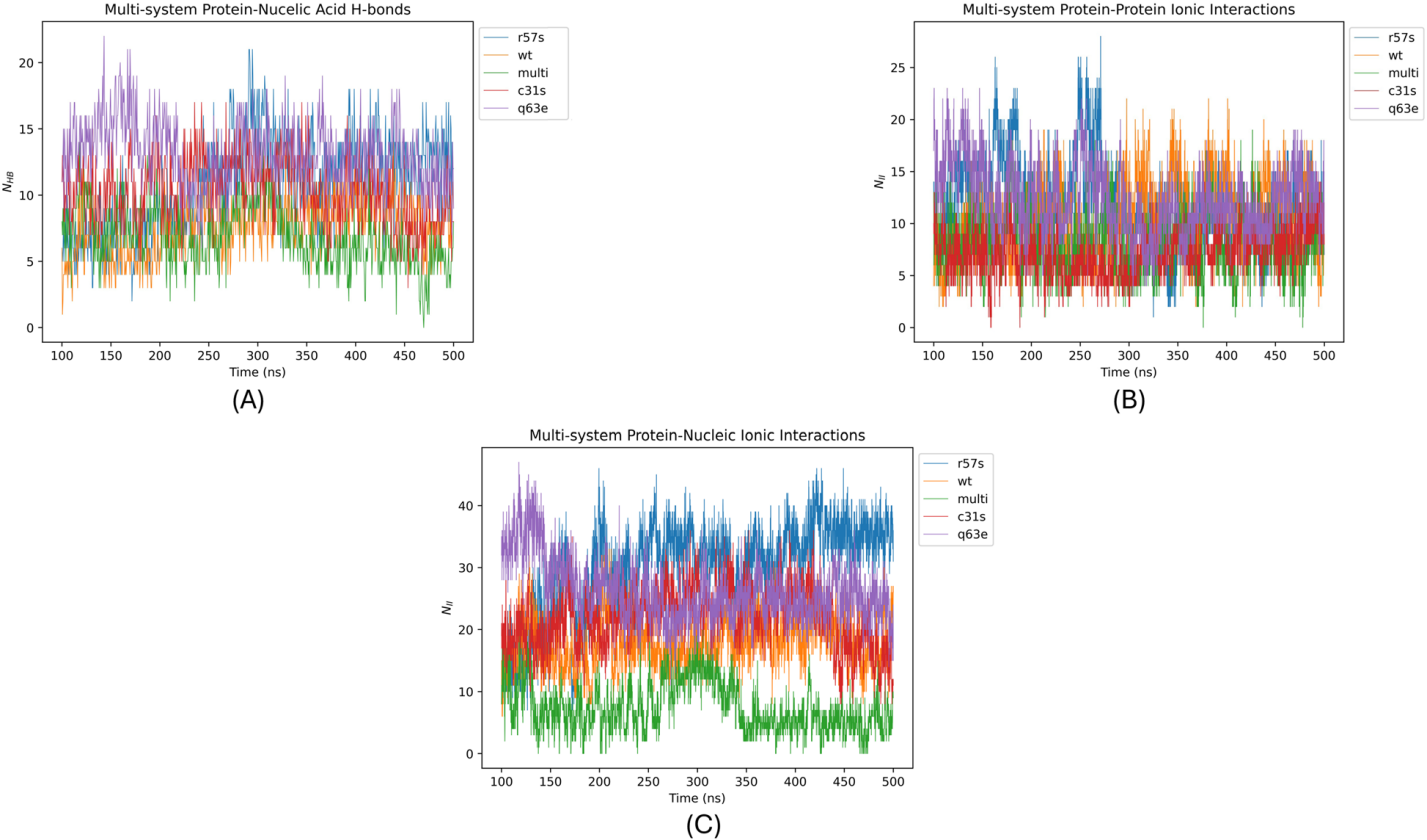
**A-C** Change in the number and type of H-bond and ionic contacts formed between Tat-TAR. **A** Plot illustrating the change in Tat-TAR hydrogen bonds (NHB), (**B**) Tat-self ionic interactions (NII), and (**C**) Tat-TAR NII of the TatWt system (orange) and variant systems TatC31S (red), TatQ63E (purple), TatR57S (blue), and TatC31SQ63ER57S (multi) (green) over the equilibrated phase (200-500ns) of the molecular dynamic simulation trajectories. Figure 8A indicates that the Tat variant systems TatC31S (red), TatR57S (blue), and TatQ63E (purple), except for TatC31S/R57S/Q63E (mulit) (green), showed similar values compared to the TatWt system (orange). In Figure 8B, NII for variant systems TatR57S (blue) and TatQ63E (purple) attained higher mean and deviation values, conversely, the C31S (red) and TatC31SQ63ER57S (mulit) (green) system had lower mean values, in comparison to the TatWt values (orange). Figure 8C indicates that the NII for the Tat variant system TatC31S (red) and TatQ63E (purple) values were higher whereas the TatC31SQ63ER57S (multi) (green) was lower compared to the TatWt system (orange)

The variations in hydrogen bonding between the protein and the respective ligand throughout a simulation contribute to the understanding of the specificity of the interaction [74] within the Tat-TAR complex. Introducing amino acid substitutions alters the potential number of hydrogen acceptors and donors, as well as the bonds (H-bonds) between the protein and the ligand. This may influence the binding stability and binding affinity of the ligand [75, 76]. From the analysis of the H-bonds using the AMIA pipeline, the number of bonds during the equilibrated region of the simulation noticeably increased in the Tat variants systems TatC31S, TatR57S and TatQ63E, except for TatC31S/R57S/Q63E, which showed similar values compared to the TatWt system. This is further supported by the mean and standard deviation values for TatWt of 7 ± 2 N_*HB*_ with the corresponding values of 10 ± 2 N_*HB*_ for TatC31S, 11 ± 4 N_*HB*_ for TatR57S, 13 ± 3 N_*HB*_ for TatQ63E and 7 ± 2 N_*HB*_ for TatC31SQ63ER57S (Fig. 8A).

Ionic interactions (II) arise directly from the differences in charge between two groups of opposite charge. These interactions significantly contribute to the stability of the protein structure as well as the stability of the protein-ligand interaction [77]. The substitution of a positively or negatively charged residue within a specific local region of the protein conformation may directly impact the dynamics, not only within the Tat protein but potentially influencing the Tat-TAR interactions as well [78]. The protein-self II (N_*II*_) for variant systems TatR57S and TatQ63E attained a higher mean and deviation values of 11 ± 4 N_*II*_ and 12 ± 3 N_*II*_, respectively. Conversely, the C31S and TatC31SQ63ER57S system had lower mean values of 8 ± 2 N_*II*_ and 9 ± 3 respectively, in comparison to the TatWt value of 10 ± 4 N_*II*_ (Fig. 8B).

The Protein-Nucleic Acid ionic interactions (N_*II*_) indicates that the TatWt system had a mean value of 16 ± 5 while the mutant system values were higher with TatC31S having 21 ± 5, TatR57S at 28 ± 8, TatQ63E at 26 ± 5 whereas the TatC31SQ63ER57S was lower at 8 ± 4 (Fig. 8C).

Analysing the binding free energy calculated using MMPBSA (Table 2), the TatQ63E followed by TatR57S showed the highest overall binding free energy (∆G TOTAL of -349.2 ± 10.4 kcal/mol and -290.0 ± 9.6 kcal/mol) compared to the three variant systems tested. This was contributed by higher van der Waals energy -290.0 ± 9.6 kcal/mol and ESURF (-23.7 ± 1.4 kcal/mol) for TatQ63E while for Tat R57S the highest energy contributors were ELE (-757.5 ± 26.7 kcal/mol) and non-polar energies (-906.4 ± 27.8 kcal/mol). TatR57S mutant system also recorded the largest positive EGB (783.6 ± 29.3 kcal/mol) and polar (761.4 ± 29.3 kcal/mol) energies compared to all the systems tested. Furthermore, the TatC31SQ63ER57S-multi system showed the lowest binding free energy (∆G TOTAL of -235.4 ± 32.5 kcal/mol) due to lower EGB (582.8 ± 31.3 kcal/mol) and polar (566.5 ± 29.3 kcal/mol) contribution energies. Compared to all the mutant systems, the TatWt recorded higher binding free energy compared to mutant systems TatC31S and the TatC31SQ63ER57S-multi system.

**Table 2:** MMPBSA analysis comparing TatWt and variant systems (TatC31S, TatQ63E, TatR57S and TatC31SQ63ER57S-multi), performed for 1001 frames for the last 100ns

**Energy component**	**Average (kcal/mol)**				
	**TatWt**	**TatC31S**	**TatQ63E**	**TatR57S**	**TatC31S/Q63E/R57S**
**∆G VDW**	-118.5 ± 12.5	-114.3 ± 15.1	-169.1 ± 13.4	-148.9 ± 12.3	-118.7 ± 20.3
**∆G ELE**	-644.9 ± 24.0	-648.7 ± 19.8	-611.1 ± 20.2	-757.5 ± 26.7	-565.5 ± 27.9
**∆G EGB**	655.0 ± 24.6	660.4 ± 21.0	629.3 ± 22.5	783.6 ± 29.3	582.8 ± 31.3
**∆G ESURF**	-15.6 ± 1.5	-16.1 ± 1.6	-23.7 ± 1.4	-22.2 ± 1.4	-16.3 ± 3.1
**∆G non-polar**	-763.4 ± 28.3	-763.0 ± 20.7	-780.2 ± 20.2	-906.4 ± 27.8	-684.2 ± 41.8
**∆G polar**	639.5 ± 14.0	644.3 ± 21.1	605.6 ± 22.3	761.4 ± 29.3	566.5 ± 29.3
**∆G TOTAL**	-247.9 ± 27.7	-237.4 ± 36.2	-349.2 ± 10.4	-290.0 ± 9.6	-235.4 ± 32.5

Abbreviations: *VDW* Van Der Waals forces, *ELE* Electrostatic force of attraction, *EGB* polar contribution to solvation energy by GB/PB method, *ESURF* non-polar contribution to solvation energy using solvent accessible surface area (SASA)

## Discussion

In this study, we employed molecular modelling to produce a reliable 3D structure of TatWt that was used to generate Tat variant structures. Subsequent molecular docking and dynamic simulations studies were conducted to investigate the potential impact of subtype C-specific amino acid substitutions on Tat-TAR binding. Several key findings surfaced in this study which are divided firstly into the molecular docking studies and secondly molecular dynamics simulation analysis. Docking studies unveiled that TatWt had the highest predicted binding affinity for TAR among subtype C-specific Tat variant structures, while the introduction of single-point subtype C-specific amino acid substitutions led to decreased binding affinity. Notably, TatR57S exhibited the most substantial reduction, attributed to fewer intermolecular interactions and increased structural flexibility, elucidating its lower predicted binding affinity relative to TatWt. However, the predicted binding free energy as reported by MD analysis indicated that the TatQ63E and TatR57S had the strongest affinity for the TAR element as compared to the TatWt. Furthermore, the TatWt had a slightly higher binding free energy for the TAR element compared to the TatC31S and the TatC31S/Q63E/R57S system.

First, our molecular docking analysis revealed that TatWt exhibited the highest predicted binding affinity for TAR, consistent with our previous computational study [34] and findings reported by other computational work [33]. The introduction of single-point amino acid substitutions resulted in a lower predicted binding affinity, with TatR57S showing the greatest reduction in binding compared to TatWt. This aligns with previous studies suggesting that amino acids within the range of 48-58 are within the TAR binding domain, and therefore fundamental for TAR interactions [79]. Considering that Arginine is a much larger and positively charged amino acid than Serine (neutral), the alteration of this amino acid may influence the structure of Tat and the electrostatics within this region, and in so doing affect the interaction with TAR.

Interestingly, the introduction of only TatC31S resulted in the smallest decrease in predicted binding affinity compared to TatWt. This observation may be attributed to the fact that the C31S amino acid variant is situated outside the recognized active TAR-interacting residues [50]. This finding aligns with prior experimental studies wherein mutations within the Tat N-terminal domain (C34S and C37W) led to a minor decrease in TAR binding affinity [80]. This is in contrast with experimental studies indicating that mutations within the basic domain result in significant decreases in TAR binding [20, 50, 81]. Hence, this further supports the notion that amino acids outside the basic domain may not notably influence Tat-TAR binding. The Tat Q63E amino acid variant is present in the N-terminal domain of the Tat protein within subtype C. Following the C31S amino acid variant, the Q63E amino acid variant displayed the second-lowest reduction in Tat-TAR binding affinity. Q63E is also situated outside the basic domain of the Tat protein, known for its interaction with TAR. Consequently, its impact on Tat-TAR binding may be limited.

However, molecular docking primarily focuses on predicting the binding mode and affinity of ligands to a receptor at a single static conformation, while MD simulations capture the dynamic behaviour and interactions of molecules over time. Therefore, to characterize the influence of single-point mutations on Tat-TAR binding, we employed MD analysis. All the MD parameters demonstrated that the TatWt protein was more stable compared to the mutant structures, however we postulate that reduced flexibility reduces surface contacts and affinity for the TAR element as seen with the interaction results. Similarly, analysing the binding free energy results, the TatQ63E followed by TatR57S showed the highest overall binding free energy compared to the TatWt and to the other variant systems tested. This was contributed by higher van der Waals energy for TatQ63E while for Tat R57S the highest energy contributors were ELE and non-polar energies. The TatR57S mutant system also recorded the largest positive EGB, and polar energies compared to all the systems tested. Furthermore, the TatC31SQ63ER57S-multi system showed the lowest binding free energy due to lower EGB and polar contribution energies. Compared to all the mutant systems, the TatWt recorded slightly higher binding free energy compared to mutant systems TatC31S and the TatC31SQ63ER57S-multi system.

The binding free energies obtained from MD simulations are in contrast with the results of molecular docking. While the molecular docking analysis reported the highest docking score for TatWt and the lowest for TatR57S, the MMPBSA calculations suggest that TatQ63E and R57S exhibit the highest predicted binding energies over time possibly due to the higher conformational flexibility of the binding site.

On a biological basis, the introduction of specific mutations in functionally important positions of the Tat protein may significantly influence its predicted binding to TAR over time. In this context, we hypothesize that (1) single mutations at positions within the Arginine and Glutamine rich regions of the Tat proteins are crucial for TAR interaction and (2) multiple amino acids may function together as a network contributing to TAR binding. To our knowledge, this is the first study to evaluate single amino acid changes from Tat subtype B to amino acids of Tat subtype C. Previous investigations using single amino acid mutations introduced the amino acid Alanine using site-directed mutagenesis [20], which is a well-known amino acid used to maintain overall protein structure while evaluating the functional role of specific amino acids or amino acid positions. Therefore, creating subtype C-specific single mutations may significantly affect the structure of the Tat protein within fundamental domains over time, potentially explaining the disparate findings when comparing our molecular docking results to the MD analysis.

Thus, we posit that creating single point mutations at these positions in isolation may have significant effects on Tat-TAR interaction. Supporting this notion, among all Tat variants investigated, TatR57S consistently yielded higher RMSD, RMSF, Rgyr, and PCA statistical values compared to the TatWt system. This suggests that the introduction of R57S results in the Tat protein exhibiting greater protein flexibility and conformational changes when interacting with TAR compared to Tat with R57 (TatWt). In the R57S system, TAR was observed to exhibit greater stability compared to TAR in the TatWt system, indicating that R57S causes more conformational changes in the Tat structure but increased stability in the TAR structure. Similarly, Q63E showed higher values for RMSD, RMSF, Rgyr, and PCA, indicating increased flexibility of the Tat protein during interaction with TAR. This collectively suggests that alteration of single amino acids at these specific positions may significantly alter the binding dynamics to TAR.

Additionally, within these regions of the Tat protein, other amino acids may collectively play a structural role together with our investigated variants in binding TAR. It is known that in addition to the amino acids we investigated within the Arginine/Glutamine region, other amino acid variants are present in Tat subtype B compared to Tat subtype C. Therefore, multiple amino acids within these pockets/domains may contribute to the structural capacity in binding TAR, and thus, creating these single amino acid variants within this region of the Tat protein may have affected the structural capacity of surrounding amino acids, significantly influencing the interaction with TAR.

Further supporting the idea that positions 57 and 63 are fundamental in TAR interaction, more negligible effects were noted for TatC31S, another single introduced amino acid mutation. However, the effects in terms of binding via both molecular docking and MD analysis were negligible. This suggests that amino acids outside of the Arginine-Glutamine regions may not play as significant a role in TAR interaction. Furthermore, the C31S amino acid variant also induced greater Tat flexibility and conformational changes in the Tat protein when interacting with TAR, as compared to TatWt. However, in the R57S system, the TAR structure exhibits increased stability and thus, improved binding, while in the C31S system, decreased stability is observed in the TAR structure, resulting in decreased binding. Consequently, both the Tat and TAR systems display decreased stability, potentially explaining the limited impact of C31S amino acid variation on Tat-TAR binding. This suggests that C31S may not play a significant role in Tat-TAR binding and subsequently viral transactivation and transcription. Instead, it may function in other mechanisms related to the development of HIV-1 neuropathogenesis. Specifically, the discyteine motif in Tat (C30C31) is notably seen as a chemokine chemoattractant of infected cells into the CNS [82], subsequently leading to increased neuroinflammation [83] and neuronal damage [84].

Furthermore, we observed a consistent trend in both molecular docking and binding free energies for TatWt, representing Tat subtype B, and Tat C31S/R57S/Q63E, which more closely resembles subtype C. Collectively, our findings suggest that the closer the Tat resembles subtype C, the lower the predicted binding affinity of TAR and more stable the dynamics of the protein in comparison to Tat subtype B. This is in line with previous investigations both on a computational [34] and experimental level that report that Tat subtype C has a lower affinity for TAR and subsequently a lower level of transactivation [22]. In a previous computational study done by our group, we compared Tat-TAR binding between Tat subtype B and C [34]. However, we were not clear which of these previously identified neuropathogenic Tat amino acids may have the biggest influence on Tat-TAR binding. Therefore, the MD simulations from this study provided crucial insights into the direct contributions of each amino acid substitution to TAR binding between the Tat subtype B and subtype C.

The findings presented in this paper offer valuable insights into the potential differences in binding of TAR element to different Tat subtype C introduced variant structures and may have possible functional roles in neuropathogenic outcomes between Tat subtype B and C infections. By identifying subtype-specific sequence variations in Tat-TAR binding and their impact on downstream effects, our study opens up avenues for further investigation. In particular, previous studies have shown that when comparing the function of Tat subtype variants (e.g., Tat subtype B vs. Tat subtype C) on underlying neuropathogenic mechanisms, a greater level of neurotoxicity has been observed for Tat subtype B. Certain studies reasoned that Tat subtype B induces higher levels of neuroinflammation and neurotoxic products compared to subtype C, thereby resulting in a greater degree of neuronal damage. However, it remains unclear whether viral transactivation (Tat-TAR interaction) and subsequent replication kinetics influence this, as this aspect has not yet been investigated.

We believe that the findings presented hold clinical significance. Firstly, we offer insights into the pivotal amino acids involved in TAR interaction, which constitutes the initial step in viral transcription efficiency. Consequently, this knowledge could serve as a starting point for developing inhibitors to disrupt Tat-TAR interactions, thereby impeding viral transcription and reducing viral replication rates. Secondly, we shed light on potential factors contributing to differential clinical outcomes observed among individuals infected with various virus subtypes. This disparity may be attributed to variations in Tat-TAR interaction and, consequently, viral fitness. Thus, our findings have the potential to inform the development of precision medicine strategies tailored to individual virus subtypes, optimizing treatment efficacy. The issue of subtype-specific treatment regimens has been under investigation for many years [85, 86]. Increasingly, recent evidence suggests that HIV-1 research and treatment strategies should not adopt a one-size-fits-all approach [87, 88]. Instead, variations in subtypes should be considered in understanding (neuro)pathogenesis and devising treatment strategies. Consistent with our findings, we propose that beyond the association of drug resistance with specific subtypes [89], there is a crucial need to comprehend the functional properties of HIV-1 in (neuro)pathogenesis when comparing different subtypes.

The question regarding subtype specific treatment regimens has been a topic of investigation for many years, more and more recent evidence suggest that HIV-1 research and HIV-1 treatment strategies should not have a one size fit all approach and indeed subtype variation should eb considered in understanding (neuro)pathogenesis and treatment strategies [86]. In line with our findings, we proosed that beyond fact that subtype variation have is associated drug resistance in particular subtypes, there is also a need to understanding the functional properties of HIV-1 may in the (neuro)pathogenesis when comparing subtypes.

To the best of our knowledge, no study has yet provided molecular validation regarding which HIV-1 subtype may have a higher binding affinity for TAR [20]. Previous investigations in this area have primarily focused on introducing mutations, typically alanine substitutions, to disrupt specific wild-type amino acid side chains and evaluate their impact on TAR binding. In contrast, our study specifically examined the influence of subtype C-specific amino acid variations on TAR binding. Future research should employ molecular techniques to experimentally validate the role of subtype-specific sequence variations in Tat-TAR binding and elucidate their potential implications for pathogenesis and neuropathogenesis. By doing so, we can gain a deeper understanding of the molecular mechanisms underlying the differential outcomes observed in different HIV-1 subtypes and pave the way for the development of targeted interventions.

## Limitations

The results of this study do support the findings from previous literature, however there are some limitations to the study. Firstly, molecular modelling can result in the generated protein model having the incorrect side chain placement, potentially affecting protein-protein interactions. However, this issue can be mitigated using additional side chain optimization tools. Furthermore, not considering the protomeric state of the protein structures during modelling could result in incorrect surface residue placement for interaction calculations.

Secondly, the findings from the molecular docking study should be interpreted in light of relevant limitations. The lack of biological information and homologous template protein structure complexes to guide the docking process may be a confounding factor in accurately predicting interactions between the TAT and TAR elements. Further, the type of docking tool used may influence the results. In this study, we utilized HDOCK, an ab initio docking program [49]. When using an alternative docking tool, HADDOCK, we noted differences in the predicted binding score (Supplementary file). This may be due to the fact that HADDOCK uses data-driven approaches that integrate information derived from biochemical, biophysical, or bioinformatics methods to enhance sampling, scoring, or both [90]. According to the official evaluation by CAPRI, the HDOCK protocol emerged as the top docking server for predicting the structure of multimeric proteins in the CASP13-CAPRI experiment [4]. Further, we selected HDOCK for this study because it was utilized in our primary in silico study to perform a direct comparison to our previous results [34]. This current study served as a follow-up to our original analysis, enabling a clearer comparison of our findings since both studies used the same docking tools. Despite the differences, a common trend was observed with both tools: the R57S mutation resulted in the lowest predicted binding affinity. This further supports that this particular amino acid position is fundamental in TAR binding.

Furthermore, the inclusion of binding free energies to include solvation and accounting for complex conformational entropy might in part address these limitations by estimating accurate binding free energies between the Tat protein and TAR element. The MMPBSA is widely applied as an efficient and reliable free energy simulation method to model molecular recognition, such as for the protein-ligand binding interactions [91], as done in this study. The MMPBSA also analyses energy contributions by free energy decomposition, giving more information into the energetics of the system being investigated. Finally, despite the computational efficiency and low cost of MMPBSA used in this study, MMPBSA does not include conformational entropy and do not consider the free energy and number of water molecules in the binding site. This can be ameliorated with the use of alchemical free energy permutation methods in future studies although being computationally expensive they do provide very accurate binding free energies by considering different intermediate states of the protein complex and free ligand.

## Conclusion

In this study, we employed homology modelling to generate an accurate 3D structure of the Tat protein using multi-conformational states of the template structure and utilized molecular docking and MD analysis to investigate the influence of Tat subtype C-specific mutations on Tat-TAR interaction. Docking studies revealed that TatWt exhibited the highest predicted binding affinity for TAR among subtype C-specific Tat variant structures. However, the introduction of single-point subtype C-specific amino acid substitutions R57S, Q63E, C31S resulted in decreased binding affinity. Notably, TatR57S variant structure showed the weakest binding affinity to Tat based on docking score, supported by fewer intermolecular interactions. However, the predicted binding free energy predictions as reported by MD analysis indicated that TatQ63E and TatR57S had the greatest predicted binding affinity for the TAR element compared to TatWt. Interestingly, this coincided with increased flexibility of Tat Q63E and R57S protein that increased TAR binding and the number of interactions. The difference in molecular docking and simulation results should be interpreted with caution until additional validation is performed. We do, however, hypothesize that the Arginine/Glutamine basic domain (residues 48-58, and 60 -72) that contain R57S and Q63E is fundamental for TAR interaction, and there is the potential for several amino acids within these domains/regions to function collectively in maintaining Tat structure for binding TAR. The findings of this study carry clinical significance, as these specific amino acids and their positions could serve as potential target sites for the design of site-specific Tat-TAR inhibitors based on the variant profile of Tat subtype. Additionally, we offer insights into potential reasons for lower viral fitness when comparing different subtypes, which may ultimately enhance our understanding of the differential clinical outcomes observed among individuals infected with these subtypes.

To further understand the outcomes of this study, future experimental studies involving binding assays and transcriptional assays should be conducted. We therefore provide insight into By investigating the influence of subtype-specific sequence variations, we can gain deeper insights into the molecular mechanisms underlying the neurological effects of different HIV-1 subtypes in PLWH.

### Supplementary Information

Supplementary Material 1.

## Data Availability

No datasets were generated or analysed during the current study.

## Abbreviations

AMIA: Automated Mutation Introduction and Analysis
CNS: Central nervous system
DOPE: Discrete Optimized Protein Energy
ELE: Electrostatic force of attraction
EM: Energy minimization
fs: Femtosecond
HIV-1B: HIV-1 subtype B
HIV-1C: HIV-1 subtype C
HAND: HIV-associated neurocognitive disorders
H-bonds: Hydrogen Bonds
LTR: Long terminal repeat
MD: Molecular Dynamic
MMPBSA: Molecular Mechanics Poisson-Boltzmann Surface Area
ns: Nanoseconds
NMDAR: N-methyl-D-aspartate receptor
ps: Picoseconds
PCA: Principal Component Analysis
pcs: Principal components
PLIP: Protein-Ligand Interaction Profiler
RMSF: Root Mean Square Fluctuation of the protein residues
Rg/Rgyr: Radius of Gyration
SASA: Solvation energy using solvent accessible surface area
SAVE: Structure Analysis and Verification Server
QA: Subsequent Quality Assessment
tatwt: Tat subtype B
TAR: Trans-activation response element
Tat: Transactivator or transcription
VDW: Van Der Waals forces
Wt: Wild type
