# Supplementary material for: Impact of subtype C-specific amino acid variants on HIV-1 Tat-TAR interaction: insights from molecular modelling and dynamics
Source: Virol J. 2024 Jun 25;21:144. doi: 10.1186/s12985-024-02419-6 (PMC11202254; doi:10.1186/s12985-024-02419-6)
Supplement: Supplementary file 1 — Supplementary Material 1. [file 12985_2024_2419_MOESM1_ESM.docx]

**Supplementary Table 1: Docking scores using HADDOCK webserver**

| **Tat variant** | **Docking Score** | **Percentage difference in predicted binding energy** |
| --- | --- | --- |
|  |  |  |
| TatWt | -77.7 +/- 6.8 | None |
| TatC31S | -82.2 +/- 9.5 | 5.8% increase |
| TatQ63E | -77.7 +/- 8.9 | 0% |
| TatC31S/R57S/Q63E | -66.7 +/- 4.5 | 14.16% decrease |
| TatR57S | -63.9 +/- 4.7 | 17.8% decrease |


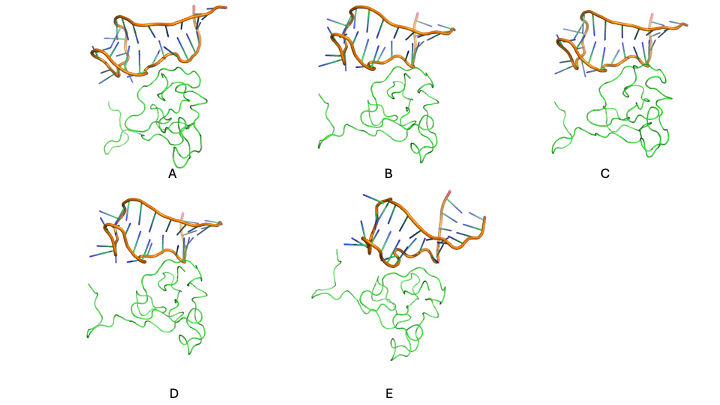


**Supplementary Figure 1:** Binding pose for Tat-TAR docking using HADDOCK webserver. A: TatWt_TAR, B: TatC31S_TAR, C: TatR57S_TAR, D: TatQ63E_TAR, E: TatC31S/R57S/Q63E_TAR
